# P-121. Brucellosis outbreak in northwestern Tajikistan in 2023: a matched case-control study

**DOI:** 10.1093/ofid/ofae631.327

**Published:** 2025-01-29

**Authors:** Emomali Qurbonov, Jamila Silemonshoeva, Zulfiya H Tilloeva, Rajabali Sharifov, Salomudin Yusufi, Dilyara Nabirova, Roberta Horth

**Affiliations:** Central Asia Advanced Field Epidemiology Training Program, Khujand, Sughd, Tajikistan; Central Asia Field Epidemiology Training Program, Khujand, Sughd, Tajikistan; City Disinfection Station, Dushanbe, Republic of Tajikistan, Dushanbe, Dushanbe, Tajikistan; Central Asia Region FETP, Dushanbe, Dushanbe, Tajikistan; Ministry of Health, Dushanbe, Dushanbe, Tajikistan; CDC Central Asia office, Almaty, Almaty, Kazakhstan; US Centers for Disease Control and Prevention, Dulles, Virginia

## Abstract

**Background:**

A sharp increase in brucellosis incidence was observed in northwestern Tajikistan (from 1.0/100,000 people in 2022 to 32,7/100,000 by May 2023). Most cases, 82% (84/103), were from the same village (population=10,712). We investigated to identify risk factors and mitigate disease.

Epidemic curve of monthly cases of brucellosis from January to September 2023, Farob village, Panjakant district, Sughd region (n=123).

**Figure:**
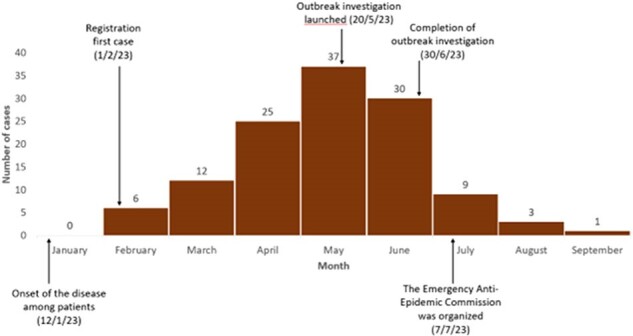

**Methods:**

Using a case-control design, we conducted face-to-face interviews and collected blood from May-July 2023. Cases were the first person in a household diagnosed with brucellosis in February-June 2023 with positive Huddleson reaction test and Wright antibody titers ≥1/160. Two controls were selected for each case (neighbors from different households matched by age and sex). Controls testing positive were excluded (13%). We conducted conditional multivariable logistic regression to assess odds of brucellosis expressed as adjusted odds ratio (AOR) and 95% confidence intervals (CI).

Multivariate analysis of human brucellosis risk factors, Farob village, Panjakent district , Tajikistan, 2023

**Figure:**
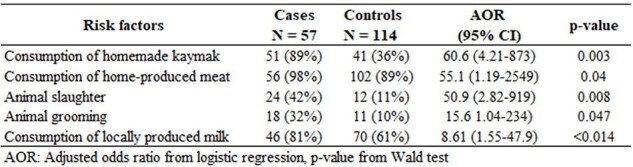

**Results:**

Of 84 cases, 57 (58%) met inclusion criteria. Of which, 68% were 15-44 years old and 44% were male. Cases peaked in May. Common symptoms were joint pain (95%), fever (84%), weakness (72%), and night sweats (65%). All cases and 94% of controls (n=114) had livestock (mostly cattle, sheep or goats); no animals had not been vaccinated < 5 years. A greater proportion of cases than controls had consumed homemade kaymak (clotted cream from unpasteurized milk) (89% vs 36%), home-produced meat (98% vs 82%) or neighborhood-produced milk (81% vs 61%); or engaged in animal slaughter (42% vs 11%) or animal grooming (32% vs 10%). Brucellosis was associated with consumption of homemade kaymak (AOR=60.6 [CI=4.2-873.0], p=0.003), homemade meat (55.1 [1.2-2,549.0], p=0.040), neighborhood milk (8.6 [1.6-47.9], p=0.014), animal slaughter (50.9 [2.8-919.0], p=0.008) or animal grooming (15.6 [1.1-234.0], p=0.047).

**Conclusion:**

Contact with unvaccinated livestock or consumption of their products was a key contributor to this outbreak. Cases were likely higher than reported. Following our investigation, an education and vaccination was carried out and no more brucellosis cases were reported after August 2023.

**Disclosures:**

**All Authors**: No reported disclosures

